# B1 lymphocytes develop independently of Notch signaling during mouse embryonic development

**DOI:** 10.1242/dev.199373

**Published:** 2021-08-09

**Authors:** Nathalia Azevedo Portilho, Rebecca Scarfò, Elisa Bertesago, Ismail Ismailoglu, Michael Kyba, Michihiro Kobayashi, Andrea Ditadi, Momoko Yoshimoto

**Affiliations:** 1 Center for Stem Cell Research and Regenerative Medicine, Institute for Molecular Medicine, McGovern Medical School, The University of Texas Health Science Center at Houston, Houston, TX 77030, USA; 2 San Raffaele Telethon Institute for Gene Therapy, IRCCS San Raffaele Scientific Institute, Milan 20132, Italy; 3 Lillehei Heart Institute and Department of Pediatrics, University of Minnesota, Minneapolis, MN 55455, USA; 4 Genetics and Development Graduate Program, University of Texas Southwestern Medical Center, Dallas, TX 75390, USA

**Keywords:** B1 cells, B2 cells, Lymphopoiesis, Notch signaling, Hematopoietic stem cells, Mouse embryonic stem cells, Mouse embryonic development

## Abstract

B1 lymphocytes are a small but unique component of the innate immune-like cells. However, their ontogenic origin is still a matter of debate. Although it is widely accepted that B1 cells originate early in fetal life, whether or not they arise from hematopoietic stem cells (HSCs) is still unclear. In order to shed light on the B1 cell origin, we set out to determine whether their lineage specification is dependent on Notch signaling, which is essential for the HSC generation and, therefore, all derivatives lineages. Using mouse embryonic stem cells (mESCs) to recapitulate murine embryonic development, we have studied the requirement for Notch signaling during the earliest B-cell lymphopoiesis and found that *Rbpj*-deficient mESCs are able to generate B1 cells. Their Notch independence was confirmed in *ex vivo* experiments using *Rbpj*-deficient embryos. In addition, we found that upregulation of Notch signaling induced the emergence of B2 lymphoid cells. Taken together, these findings indicate that control of Notch signaling dose is crucial for different B-cell lineage specification from endothelial cells and provides pivotal information for their *in vitro* generation from PSCs for therapeutic applications.

This article has an associated ‘The people behind the papers’ interview.

## Abstract (precis)

**Summary:** During development, the first B lymphocytes are produced in the absence of Notch signaling and, thus, independently from HSCs.

## INTRODUCTION

B1 cells are a subtype of B lymphocyte that colonize body cavities and mucosa. Unlike conventional B lymphocytes (B2 cells), B1 cells are able to mount a rapid response to antigenic stimuli, and they do not depend on T-cells to differentiate into effector antibody-producing plasma cells (Martin et al., 2001). Studies in mice suggest that B1 cells generate many of the natural antibodies that are present at mucosal sites, the production of which does not depend on prior immunization (Smith and Baumgarth, 2019). In particular, B1 cells produce antibodies that are both antimicrobial, in particular against polysaccharide antigens of microorganisms, and homeostatic, i.e. reactive with auto-antigens (Baumgarth, 2017). Moreover, they display very peculiar features, such as phagocytic ability, antigen presentation and induction of proinflammatory T-cell differentiation (Aziz et al., 2015). For these reasons, they are often referred to as innate-like B-cells and play a pivotal role in innate immunity and tissue homeostasis.

B1 cells are generated predominantly during fetal life and persist throughout adulthood thanks to their self-renewal ability (Baumgarth, 2017; Kobayashi et al., 2020). Because fetal liver (FL) is the primary source for B1a cell production, as shown by transplantation assays (Hardy and Hayakawa, 1991), it has been assumed that FL hematopoietic stem cells (HSCs) are the main provider of postnatal B1a cell pool in the peritoneal cavity. Indeed, recent papers have shown FL HSC-derived B1a cell repopulation (Beaudin et al., 2016; Kristiansen et al., 2016); however, we and others have demonstrated that highly purified FL HSCs failed to repopulate B1a cells upon transplantation (Ghosn et al., 2016; Kobayashi et al., 2019). These contradictory reports led us to investigate the HSC dependency of B1 cell emergence, because understanding their exact origin is crucial for designing protocols for their *in vitro* generation from pluripotent stem cells (PSCs). Given that B1a cells are not replenished after bone marrow transplant, PSCs would serve as a unique source of B1 cells. In order to determine whether B1 cells have a truly HSC-independent origin, we studied the requirement of Notch signaling for their emergence. It is well accepted that Notch signaling is indispensable for the generation of HSC from hemogenic endothelial cells through the endothelial-to-hematopoietic transition (EHT) (Kumano et al., 2003; Robert-Moreno et al., 2005). However, the role of Notch signaling in HSC-independent hematopoietic programs, such as those generating tissue-resident immune cells (e.g. microglia, epidermal γδT-cells and B1a cells), remains unclear (Dzierzak and Bigas, 2018). Experiments in different animal models have shown that the specification of primitive as well as erythroid-myeloid progenitors (EMPs) in the yolk sac (YS) is largely unaffected in the absence of Notch signaling (Kumano et al., 2003; Hadland et al., 2004; Robert-Moreno et al., 2005; Bertrand et al., 2010), but its requirement for fetal B-lymphocytes is unknown. Using mouse embryonic stem cells (mESCs) to recapitulate murine embryonic development, we found that Notch-deficient (*Rbpj*^−/−^) mESCs are able to generate B1 cells. Our results indicate that Notch signaling is dispensable for the emergence of fetal B1 cells and that its fine control induces B2 cell specification.

## RESULTS AND DISCUSSION

We first validated that *Rbpj^−/−^* mESC hematopoietic differentiation faithfully phenocopies what has been reported in several animal models defective for Notch signaling (Kumano et al., 2003; Hadland et al., 2004; Robert-Moreno et al., 2005; Bertrand et al., 2010). Both day 3.25 and day 5.5 Flk1^+^ cells from *Rbpj^−/−^* embryoid bodies (EBs) produced a higher number of primitive erythroid colony-forming cells (EryP-CFCs) than *Rbpj^+/−^* mESCs (Fig. S1A-H). Within the day 5.5 Flk1^+^ fraction, nearly half (45.7%) were already CD41^+^ of *Rbpj^−/−^* cells, compared with the relatively small fraction observed from *Rbpj^+/−^* mESCs (8.2%) (Fig. S1I,J). The CFC potential of day 5.5 Flk1^+^ cells, including EryP-CFCs, segregated to the CD41^+^ fraction in both lines (Fig. S1K), confirming that the clonogenic potential measured directly at day 5.5 is of primitive origin. Altogether, these results indicate that Notch signaling is required for the proper termination of primitive erythropoiesis in mESC differentiating cultures, as previously described using mouse embryos (Hadland et al., 2004; Robert-Moreno et al., 2007).

**Fig. 1.**
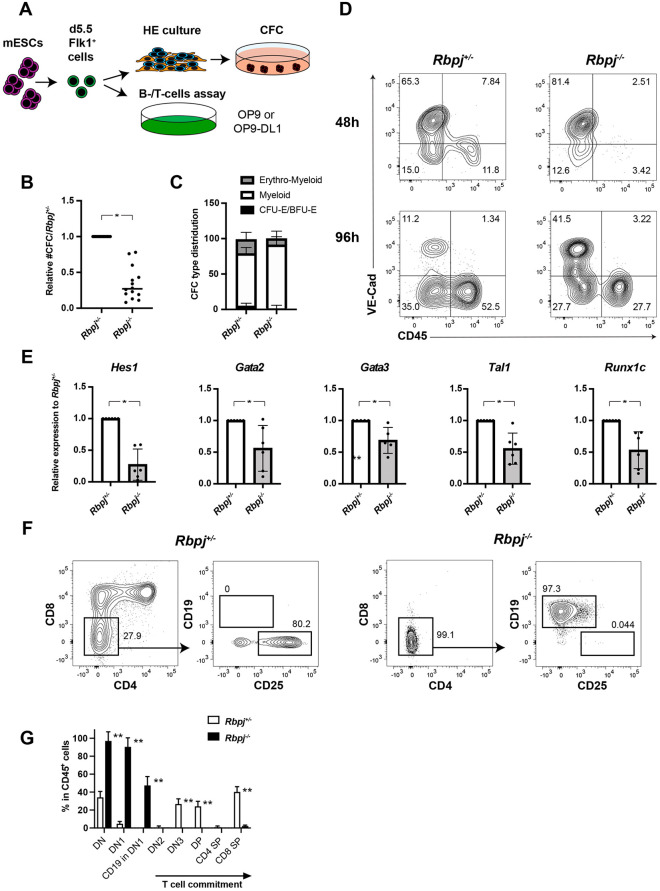
**Hematopoietic output from day 5.5 Flk1^+^ cells yielding EMP-like progenitors is reduced in the absence of Notch signaling.** (A) Experimental design. (B,C) Relative number of CFCs obtained after 96 h of EHT culture (B) and their lineage distribution (C). (D) Representative FACS analysis of VE-Cad and CD45 expression after 48 and 96 h of EHT culture. (E) qRT-PCR-based gene expression analysis in day 5.5 Flk1^+^ cells. (F) Representative FACS analysis of lymphoid markers in cells obtained from *Rbpj^+/−^* and *Rbpj^−/−^* Flk1^+^ cells cultured on OP9-DL1 for 15 days. (G) Quantification of the proportion of each T-cell stage following Flk1^+^ cell differentiation on OP9-DL1. DN, double negative for CD4 and CD8; DN1, CD44^+^CD25^−^; DN2, CD44^+^CD25; DN3, CD44^−^CD25^+^; DP, double positive for CD4 and CD8; SP, single positive. *n*>3 independent experiments. Student's unpaired *t*-test (*P<0.05, **P<0.01). Data are mean±s.d.

Next, we tested the ability of day 5.5 Flk1^+^ cells, which yield hematopoietic progenitors closely resembling EMPs (Clarke et al., 2015), to undergo EHT (Fig. 1A) and found that *Rbpj^−/−^* day 5.5 Flk1^+^ cells generated significantly fewer clonogenic progenitors (2.9-fold) (Fig. 1B), in particular of the erythroid lineage (Fig. 1C). In line with previous reports (Clarke et al., 2015), the β-globin gene expression pattern of these erythroid colonies is similar to those of cultured YS-derived EMPs (McGrath et al., 2015), as they express a lower level of *Hbb-y* compared with EryP-CFC and contain mainly adult β1-globin transcripts (Fig. S2A,B). Flow cytometric analysis for the emergence of CD45^+^ hematopoietic cells during EHT cultures confirmed the hematopoietic impairment of *Rbpj^−/−^* day 5.5 Flk1^+^ cells by marked reduction of the CD45^+^ fraction compared with their *Rbpj^+/−^* counterpart. This reduction of hematopoietic output from *Rbpj^−/−^* day 5.5 Flk1^+^ cells was already apparent after 48 h of EHT cultures (Fig. 1D). Extending EHT cultures to 144 h resulted in even greater differences in hematopoietic output, measured as the proportion of CD45^+^ cells, CD11b^+^ myeloid and Ter119^+^ erythroid cells, as well as clonogenic progenitors in EHT cultures (Fig. S2C-E). This suggests the deficiency in hematopoietic potential observed in *Rbpj*^−/−^ day 5.5 Flk1^+^ cells is unlikely due to variability in the differentiation kinetics of the two mESC lines but is rather an outcome of the absence of Notch signaling. Gene expression analysis via qPCR revealed that known Notch targets (*Hes1* and *Gata2*), as well as pivotal hematopoietic transcription factors (*Tal1* and *Runx1c*), are significantly downregulated in day 5.5 Flk1^+^ cells (Fig. 1E). These results, in line with those obtained with chimeric *Notch1^−/−^* mice (Hadland et al., 2004), suggest *Rbpj* is not required for the generation of EMP hematopoiesis but instead alters the proliferation or survival of hematopoietic progenitors. Alternatively, day 5.5 Flk1^+^ cells, and probably ECs yielding EMPs in the mouse embryos, are heterogeneous and comprise both Notch-dependent and -independent precursors. As both *Rbp^+/−^*- and *Rbpj^−/−^*-derived Flk1^+^ cells are devoid of HSC potential (data not shown), collectively, these data indicate that the differentiation of *Rbpj^−/−^* mESCs represents a valuable tool for dissecting the Notch signaling requirement in different hematopoietic embryonic progenitors that are generated before HSC emergence.

We then investigated the lymphoid potential of *Rbpj^−/−^* ESCs. In the OP9-DL1 co-culture system (Yoshimoto et al., 2009), *Rbpj^+/−^* Flk1^+^ cells differentiated into CD4^+^CD8^+^ double-positive (DP) T cells, whereas *Rbpj^−/−^* Flk1^+^ cells showed a maturation arrest at the CD4^−^CD8^−^CD25^−^ (DN1) stage and a lineage switch into CD19^+^ B cells (Fig. 1F,G), consistent with what was observed in conditional *Rbpj*^−/−^ mice (Han et al., 2002). This is also compatible with a recent report showing that the first embryonic thymopoiesis-initiating progenitors were generated in the absence of *Rbpj* (Luis et al., 2016) but were unable to progress beyond the DN1 stage; however, this report did not assess the alternative B-cell fate. The earliest thymic T-progenitors (ETPs) in DN1 have been reported to possess both B-cell and myeloid potential (Luc et al., 2012), and it is well accepted that lineage differentiation into T- or B-cells depends on Notch signaling (Han et al., 2002; Dallas et al., 2005). Therefore, our observation of alternative *Rbpj^−/−^* B-cell specification in T-cell cultures supports a model where early lymphoid precursors are produced and switch to B-cell fate in the absence of Notch signaling.

**Fig. 2.**
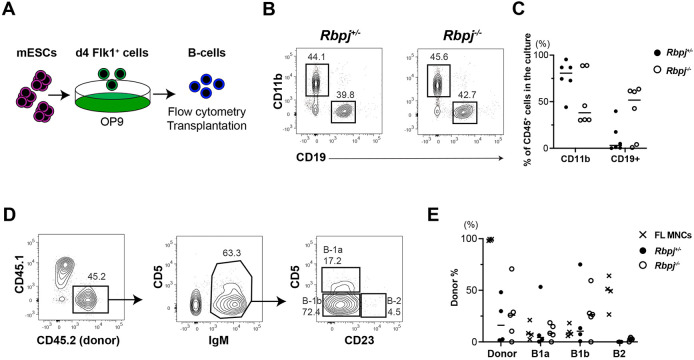
**B cells are generated from mESC-derived Flk1^+^ progenitors in the absence of Notch signaling.** (A) Experimental design. (B) Representative FACS analysis of CD11b^+^ myeloid and CD19^+^ B cells (gated as CD45^+^ cells) produced from Flk1^+^ cells on OP9 co-culture are shown. (C) The percentages of CD11b^+^ and CD19^+^ cells among CD45^+^ cells in the mESC culture with OP9 are shown (*n*=6). (D) Representative FACS analysis of donor-derived cells found in the peritoneal cavity of NSG mice transplanted with *Rbpj^−/−^* mESC-derived B progenitors. (E) Percentage of donor-derived CD45^+^ cells in the recipient peritoneal cavity after *Rbpj^+/−^*- and *Rbpj^−/−^*-derived B-cell transplantation. (*Rbpj^+/−^*, *n*=4; *Rbpj^−/−^*, *n*=5). As a control, FL MNCs were transplanted into NSG mice (*n*=4). Individual data points are shown.

Next, we cultured Flk1^+^ cells from *Rbpj^+/−^* and *Rbpj^−/−^* ESCs on OP9 stromal cells (Fig. 2A) and found that B cells were produced from *Rbpj^−/−^* ESCs, at a similar level to *Rbpj^+/−^* ESCs (Fig. 2B,C). In OP9 culture, mESC-derived Flk1^+^ cells differentiate into AA4.1^+^CD19^+^B220^+^ pre-B cells. This cell population contains a mixture of B1 and B2 progenitors (Barber et al., 2011). In order to determine their B-cell lineage potential, we transplanted *Rbpj^+/−^* and *Rbpj^−/−^* mESC-derived AA4.1^+^CD19^+^B220^+^ B-cell progenitors into sublethally irradiated newborn mice where they can differentiate into mature B-cell subsets. We previously reported that mESC-derived B-cells can engraft immunodeficient neonates and give rise to peritoneal B1 cells, but not B2 cells, *in vivo* (Lin et al., 2019), similar to E9.5 YS-derived B cells that displayed skewed B1 cell engraftment (Yoshimoto et al., 2011). Likewise, *in vitro*-derived AA4.1^+^CD19^+^ B-cell progenitors from *Rbpj^+/−^* and *Rbpj^−/−^* mESCs matured into IgM^+^CD5^+^ B1a and IgM^+^CD5^−^CD23^−^ B1b cells, but not IgM^+^CD23^+^ B2 cells, whereas control FL MNCs repopulated all B-cell subsets (Fig. 2D,E). Thus, mESCs can differentiate into transplantable B1 cell progenitors in the absence of Notch signaling. In contrast, Consistent with the failure to specify HSCs in these mESC culture conditions, mESC-derived HSC-independent B-cell progenitors are devoid of B2 lineage potential, regardless of their ability to respond to Notch signaling.

**Fig. 3.**
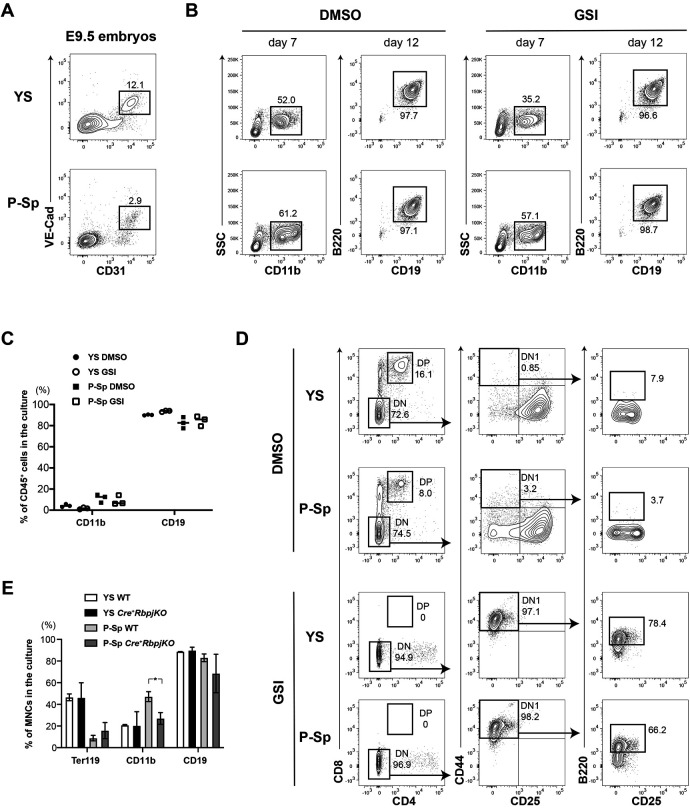
**B-cell production from ECs in the E9.5 YS and P-Sp was not affected by Notch signaling inhibition.** (A) Sorting strategy of CD31^+^VE-cad^+^ ECs from E9.5 YS and P-Sp from wild-type C57BL6 mice is shown. (B,C) Representative FACS analysis (B) and percentages of CD45^+^ cells expressing CD11b or CD19 (C) in the supernatant of ECs co-cultured on OP9 stromal cells with or without GSI (*n*=3). (D) Representative FACS analysis of E9.5 YS/P-Sp co-culture with OP9-DL1 with/without GSI (*n*=3 for each group). (E) Percentages of each blood lineage among mononuclear cells (MNCs) in the supernatant of E9.5 wild-type and *VE-Cad-Cre: RbpjKO* YS and P-Sp, co-culture with OP9 (*n*=3 for each group). Ter119/CD11b and CD19 expression were examined at day 6 and at day10 of OP9 co-culture, respectively. Student's unpaired *t*-test (*P<0.05). Data are mean±s.d.

Next, we tested the effect of Notch signaling inhibition *ex vivo* in ECs derived from wild-type mouse embryos. FACS-sorted VE-cadherin^+^ (VE-Cad-Cre^+^) ECs from E9.5 YS and para-aortic splanchnopleura (P-Sp) were plated on OP9 stromal cells with or without γ-secretase inhibitor (GSI), a small molecule that inhibits the cleavage of the Notch intracellular domain (NICD), thus preventing the activation of Notch-intracellular signaling (Souilhol et al., 2016) (Fig. 3A). CD11b^+^ myeloid and CD19^+^B220^+^ B cells were similarly produced regardless of GSI addition (Fig. 3B,C). In OP9-DL1 cultures supplemented with GSI, the T-cell development from VE-Cad-Cre^+^ ECs was arrested at the DN1 stage, where B220^+^ B cells were detected, while DP cells were produced in DMSO-treated control culture (Fig. 3D). We also confirmed the Notch-independent emergence of B-cell potential using *VE-Cad-Cre: Rbpj^f/f^* embryos (*Cre^+^RbpjKO*). *Rbpj* deletion in ECs leads to midgestation lethality, thus preventing the assessment of hematopoietic development *in vivo*. For this, we analyzed *ex vivo* the hematopoietic potential of E9.5 YS and P-Sp cells. Both *Cre^+^RbpjKO* and control cells displayed similar erythro-myeloid and B-cell production (Fig. 3E). These results indicate that, although they cannot give rise to HSCs, ECs that are unable to respond to Notch signaling harbor B-lymphoid potential. In addition, we tested the B-cell potential of the major HSC-independent hematopoietic progenitor population present at E9.5: the YS-derived EMP fraction. Sorted cKit^+^CD16/32^+^CD41^+^ EMP cells from E9.5 wild-type YS (20-25 somite pairs) did not generate AA4.1^+^CD19^+^ B cells (Fig. S2F), in line with the previous reports that only ECs have B-cell potential at E9-9.5 (Nishikawa et al., 1998; Yoshimoto et al., 2011; McGrath et al., 2015). Collectively, these results indicate that the Notch-independent B-cell specification occurs in ECs.

**Fig. 4.**
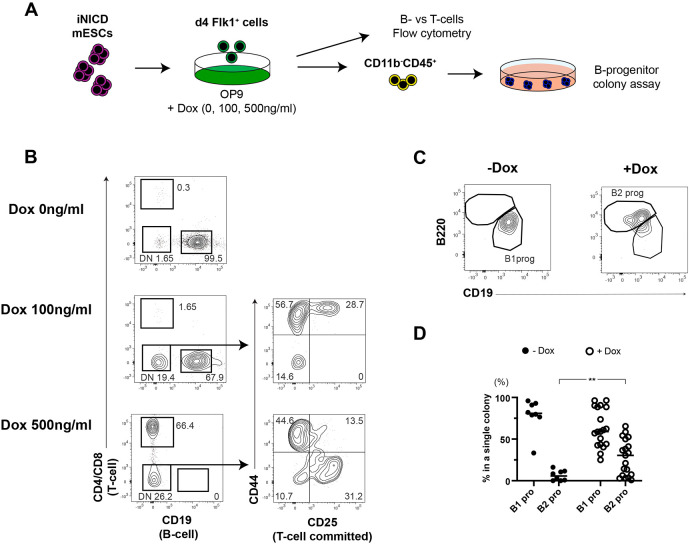
**Different doses of Notch signaling affect the lineage choice of Flk1^+^-derived cells between B1 and B2 cells.** (A) Experimental design. Flk1^+^ cells were generated from iNICD mESCs by EB formation. Sorted Flk1^+^ cells were plated on OP9 cells with the indicated dose of Dox. (B) Representative FACS plots of iNICD mESC-derived Flk1^+^ cells co-cultured with OP9 with different doses of doxycycline (*n*>3). (C) Representative FACS plots of B1 and B2 progenitors picked from a single colony. (D) Percentage of B1 and B2 progenitors present in each colony with and without 600 ng/ml doxycycline. −Dox, *n*=8; +Dox, *n*=19. Student's unpaired *t*-test (**P<0.01). Individual data points are shown.

Next, we examined the positive effect of Notch signaling on the ECs using doxycycline (Dox)-inducible NICD-overexpressing (iNICD) ESCs (Jang et al., 2015). iNICD Flk1^+^ cells were plated on OP9 with 0, 100 and 500 ng/ml Dox (Fig. 4A). In these culture conditions, Flk1^+^ cells from iNICD ESCs can produce B-lymphoid cells only in the absence of Dox. When cultured with 500 ng/ml Dox, Flk1^+^ cells can generate DP and DN CD25^+^ cells committed to the T-lymphoid lineage (Fig. 4B). Interestingly, both T and B cells were produced in the same culture when 100 ng/ml Dox was added. These results were in line with the previous report that Notch signaling dose determines B- or T-cell specification from BM hematopoietic stem/progenitor cells (Dallas et al., 2005). As we and others showed Notch signaling induced B2 potential in HSC precursors (Lu et al., 2016; Hadland et al., 2017; Kobayashi et al., 2019), we hypothesized that Notch signaling also regulates B1 and/or B2 lineage determination from Flk1^+^-derived progenitors. To test this, we used the modified B-progenitor CFC assay recently described (Montecino-Rodriguez et al., 2016; Kobayashi and Yoshimoto, 2020). Whereas B-progenitors generated *in vitro* from mESCs or *ex vivo* from mouse embryos do not express mature B1 or B2 cell markers (such as IgM, CD5 and CD23), the modified B-progenitor CFC assay distinguishes B1 and B2 progenitors based on their AA4.1, CD19 and B220 expression. Under these conditions, B1 progenitors are defined as AA4.1^+^CD19^+^B220^−^, whereas B2 progenitors display an AA4.1^+^CD19^−^B220^+^ phenotype (Montecino-Rodriguez et al., 2016; Kobayashi et al., 2019). We cultured iNICD ESC-derived Flk1^+^ cells on OP9 with different doses of Dox. After 7 days of co-culture, the resulting CD11b^−^CD45^+^ hematopoietic progenitors were plated onto methylcellulose with OP9 cells. In the absence of Dox, colonies containing uniquely B1 progenitors were generated (Fig. 4C,D), whereas colonies containing both B1 and B2 progenitors were detected only in Dox-treated cultures. Of note, colonies composed exclusively of B2 progenitors were never observed. These results suggest that the activation of Notch signaling elicited B2 potential in Flk1^+^-derived progenitors that otherwise would harbor only B1 potential. Thus, fine-tuning Notch signaling plays a role in the control B-cell lineage specification of mESC-derived Flk1^+^ cells.

Collectively, using *Rbpj^−/−^* ESCs and mouse models, we demonstrated that the development of the earliest lymphoid progenitors, in particular of B1 cells, can occur in the absence of Notch signaling. As HSC development is dependent upon Notch signaling, our results strongly support the hypothesis that at least a fraction of B1 cells arises from fetal precursors independently of HSCs. However, our data cannot rule out the possibility that other Notch-dependent hematopoietic progenitors emerging during embryonic development, including fetal HSCs, also contribute in parallel to the B-1 cell pool. These two scenarios are not mutually exclusive and, indeed, the generation of B1 cells is likely developmentally restricted to prenatal development with contribution from different waves of ontogenic progenitors (Beaudin et al., 2016; Ghosn et al., 2016; Kristiansen et al., 2016; Montecino-Rodriguez et al., 2016; Hadland and Yoshimoto, 2018; Kobayashi et al., 2019). Our results also indicate that Notch signaling is a potential regulator of the B1 versus B2 cell lineage specification in Flk1^+^-derived progenitors. As conditional *Rbpj* deletion in adult HSCs does not alter B2 cell production (Han et al., 2002), the fine-tuning of Notch signaling in B1- and B2-cell specification may occur only in progenitors during embryonic development. Further investigation is required to clarify the lineage choice of B1 versus B2 cells in fetal precursors *in vivo*. In addition, it remains to be determined whether Notch activation in ECs specifies a B1 and B2 bipotent cell, a common lymphoid or multipotent progenitor, or elicits an HSC program.

Because B1a cells are mainly fetal derived and are not replenished by adult HSCs, the conditioning regimen for HSC transplantation may induce permanent B-1 cell deficiency in humans, which may increase the susceptibility of patients to some microbial infections (Moins-Teisserenc et al., 2013). Additionally, natural IgM antibodies produced by B1 cells have protective roles against atherosclerosis and other inflammatory diseases (Aziz et al., 2015). Therefore, there is an unmet clinical need for B1 re-establishing cell therapies. Our findings are crucial for the future development of strategies for the *in vitro* generation of B1a cells from PSCs. Although robust protocols for the *in vitro* derivation of B-cells from human PSCs are still lacking, our results suggest that a stage-specific Notch signaling manipulation should be taken into consideration for the *in vitro* generation of specific lymphoid subsets.

## MATERIALS AND METHODS

### mESC maintenance and differentiation

*Rbpj^+/−^* and *Rbpj^−/−^* mESCs were a kind gift from Dr Timm Schroeder (ETH Zurich, Switzerland) (Schroeder et al., 2003). These mESC lines were maintained in serum-free/feeder-free culture conditions (SFES) supplemented with 2i (Gadue et al., 2006; Ying et al., 2008). The primitive and definitive colony-forming cells (CFCs) were produced from mESCs through EB formation, as previously reported (Irion et al., 2010; Clarke et al., 2015). Briefly, mESCs were trypsinized using TrypLE (Invitrogen) and cultured in serum-free differentiation (SFD) media in the absence of growth factors at a concentration of 1×10^5^/ml for 48 h to form EBs. At day 2 (d2), EBs were dissociated with TrypLE and reaggregated at a concentration of 2×10^5^/ml in SFD media containing human (h) VEGF (5 ng/ml), hBMP4 (1 ng/ml) and activin A (1 ng/ml) for 30 h (d3.25). For primitive hematopoietic specification, at d3.25 EBs were dissociated and Flk1^+^ cells were either isolated by FACS (using CD309 Vegfr2-APC antibody, Miltenyi Biotec) using a FACS Aria II cell sorter (Becton Dickinson) or bead sorted (using CD309 Vegfr2 Biotin followed by Anti-Biotin MACS MicroBeads, both Miltenyi Biotec) using a Miltenyi Biotec magnetic separator. Flk1^+^ cells were then reaggregated at a concentration of 2.5×10^5^/ml in 96-well low-attachment plates (Corning) in SFD containing rhVEGF (5 ng/ml) for the indicated time. For differentiation into EMP-like progenitors, at d3.25 EBs were dissociated and reaggregated at a concentration of 5×10^5^/ml in six-well tissue culture with human activin A (1 ng/ml) for 24 h, when a final concentration of hVEGF (5 ng/ml), hBMP4 (10 ng/ml) and SB431452 (6 µM, Tocris) was added to the cultures. At day 5.5, EBs were dissociated using TrypLE and Flk1^+^ cells were collected as described previously for primitive progenitors. In order to induce their EHT, d5.5 Flk1^+^ cells were aggregated overnight in 96-well low-attachment plates at a concentration of 2.5×10^5^/ml in SFD media containing mouse (m) SCF (50 ng/ml), hVEGF (5 ng/ml) and hbFGF (10 ng/ml). The next morning, aggregates were spotted on Matrigel (VWR)-coated 24-well plates for 6 h to allow adherence and then the wells were filled with SFD containing mSCF (50 ng/ml), hVEGF(10 ng/ml), hbFGF (10 ng/ml), mTPO (50 ng/ml), mIL3 (30 ng/ml), mIL6 (10 ng/ml), hIL11 (5 ng/ml), mFlt3-ligand (Flt3-L, 10 ng/ml) and hBMP4 (10 ng/ml). All cytokines were purchased from Miltenyi Biotec. Doxycycline (Dox) iNICD ESCs were maintained on mouse embryonic fibroblasts (MEFs) in DMEM with 15% FBS, MEM non-essential amino acid (Gibco), 1 mM sodium pyruvate (Gibco), 1×10^−4^ M β-mercaptoethanol and 1000 U/ml LIF (Millipore). For differentiation into Flk1^+^ cells, EBs were formed in the suspension culture (1×10^4^ cells/ml) in αMEM with 10% FBS (GenClone) and 0.5×10^−4^ M β-mercaptoethanol. On day 4 of differentiation, EBs were dissociated into single-cell suspension using 0.05% Trypsin/EDTA, and Flk1^+^ cells were sorted on FACS Aria II (BD Biosciences).

### Hematopoietic progenitor colony assay

Cells were plated in methylcellulose (STEMCELLTechnologies 03434), supplied with EPO 10 ng/ml, hIL11 10 ng/ml, mSCF 10 ng/ml, mIL6 10 ng/ml and mIL3 10 ng/ml. All cytokines were purchased from Miltenyi Biotec, except for EPO, which was purchased from Peprotech. Cultures were maintained at 37°C in 5% CO_2_ for 5-12 days.

### Real time qPCR

Total RNA was extracted with Reliaprep RNA Cell Miniprep System (Promega). RNA (100-500 ng) was reverse transcribed into cDNA using random hexamers and Oligo (dT) with ImProm-II Reverse Transcription System (Promega). qPCR was performed on FrameStar FastPlate 96 (4titude) using Fast SYBR Green Master Mix (Thermo Fisher Scientific). Expression levels were normalized to the housekeeping gene *Actb*. Oligonucleotide sequences were as follows: *Actb* (Fw, GAAGGTGACAGCATTGCTTCTGTG; Rev, CTCAGACCTGGGCCATTCAGAAT); *Hes1* (Fw, CGGCATTCCAAGCTAGAGAAGG; Rev, GGTAGGTCATGGCGTTGATCTG); *Gata2* (Fw, AAGCTGCACAATGTTAACAGG; Rev, CCTTTCTTGCTCTTCTTGGAC); *Gata3* (Fw, ATTAAATAGCTTCTATGCGCCCGGCG; Rev, ATGCATGTTGGTAGCTGGTACGCT); *Tal1* (Fw, CAGCCTGATGCTAAGGCAAG; Rev, AGCCAACCTACCATGCACAC); *Runx1c* (Fw, AGCCTGGCAGTGTCAGAAGT; Rev, GAAAGCCTGTGGTTTGCATT); *Hbb-y* (Fw, CTCTAGCTGTCCAGCAATCCTG; Rev, GCTTTCAAGGAACAGTCCAGTATTC); *Hbb-bh1* (Fw, AGTTTGGAAACCTCTCTTCTGCCCTG; Rev, TGTTCTTAACCCCCAAGCCCAAG); and *Hbb-b1* (Fw, GCTCTTGCCTGTGAACAATG; Rev, GTCAGAAGACAGATTTTCAAATG).

### Mice, tissue collection, processing and transplantation

C57BL/6 mice were purchased from Jackson Laboratory and used for breeding with transgenic mice. CD1 mice were purchased from Charles River Laboratories and used for isolating EMPs. VE-cadherin Cre (VE-Cad-Cre) mice (Chen et al., 2009) were obtained from Dr Nancy Speck (University of Pennsylvania, Philadelphia, USA). *Rbpj*-flox mice (Han et al., 2002) were obtained from Dr Tasuku Honjo (Kyoto University, Japan). *VE-Cad-Cre* mice were crossed with *Rbpj-floxed* mice, and double heterozygous mice were crossed with *Rbpj-floxed* mice to obtain *VE-Cad-Cre: Rbpj* KO embryos. Mice were timed mated, and YS and P-Sp tissues were dissected from embryonic day (E) 9.5 embryos. Embryonic tissues were staged according to somite pair counts. E9.5 YS and P-Sp were incubated in 0.125% collagenase type I (STEMCELL Technologies) at 37°C for 5-10 min and then dissociated by adding cell dissociation buffer (Thermo Fisher Scientific) with gentle manual pipetting. FL was harvested from E15.5 embryos and made into a single cell suspension by mechanical dissociation using 22G needles and syringes. Mononuclear cells (MNCs) were collected by gradient centrifugation using Histopaque 1083 (Sigma-Aldrich). For transplants, AA4.1^+^CD19^+^B220^+^ B progenitors derived from *Rbpj*^+/−^ and *Rbpj*^−/−^ mESCs cultured on OP9 were injected into the peritoneal cavity of sublethally irradiated (150 rad) NSG neonatal mice (day 2-5). E15.5 FL MNCs were injected as a positive control. Eight to 12 weeks after transplantation, the recipient peritoneal cells were analyzed for donor cell engraftment by flow cytometry. All procedures involving mice were carried out in compliance with the Animal Care and Use Committee at UTHealth or Ospedale San Raffaele (IACUC 841) and communicated to the Ministry of Health and local authorities according to Italian law.

### Flow cytometry and cell sorting

Endothelial cells (ECs) were sorted as Ter119^−^CD41^−^CD144^+^. EMP were sorted as Sca1^−^Ter119^−^CD16/32^+^cKit^+^CD41^+^. The following antibodies were used for these studies with various fluorescent color combinations: CD144 (11D4.1, BD Biosciences; 1:200), CD41 (MWReg30, eBioscience; 1:200), CD16/32 (clone 93, eBioscience; 1:200), Ter119 (TER-119 BD Biosciences; 1:200), CD8a (53-6.7, eBioscience; 1:200), CD4 (GK1.5, eBioscience; 1:200), CD45 (30 F-11, BD Biosciences; 1:200), CD25 (PC61.5, eBioscience; 1:200), CD44 (IM7, eBioscience; 1:200), CD117 (2B8, eBioscience; 1:200), Sca1 (D7, eBioscience; 1:200), CD11b (M1/70, eBioscience; 1:200), CD19 (1D3, eBioscience; 1:200), B220 (RA3-6B2, eBioscience; 1:200), CD93 (AA4.1, eBioscience; 1:200). Cells were sorted on FACSAria II (BD Biosciences).

### Co-culture with OP9 for myeloid and lymphoid differentiation

Flk1^+^ cells differentiated from mESCs or VE-Cad-Cre cells from E9.5 YS/P-Sp were plated on OP9 stromal cells for myeloid and B-cell production or on OP9 cells overexpressing delta-like 1 protein (OP9-DL1) for T cell production in αMEM supplemented with 10% FBS (GenClone), 0.5×10^−4^ M β-metcaptoethanol, mSCF (10 ng/ml), mIL7 (10 ng/ml) and mFlt-3L (10 ng/ml) as previously described (Yoshimoto et al., 2009, 2011). For Notch signaling inhibition, γ-secretase inhibitor (GSI, 10 μM, Sigma-Aldrich) or DMSO (Sigma-Aldrich) was added to the culture. Hematopoietic cells produced in culture supernatant were analyzed by flow cytometry on day 7, and on days 12-14. For iDOX-iNICD ESC differentiation, 5×10^3^-1×10^4^ Flk1^+^ cells from day 4 EBs were plated on one well of a 6-well plate confluent with OP9 cells with mSCF (10 ng/ml), mIL-7 (10 ng/ml), mFlt3L (10 ng/ml), and with 0, 100 and 500 ng/ml Dox. Hematopoietic cells present in the culture supernatant were analyzed using flow cytometry. The modified B-progenitor CFC assays were performed as previously reported (Montecino-Rodriguez et al., 2016; Kobayashi and Yoshimoto, 2020). Briefly, mESC-derived Flk1^+^ cells were cultured on OP9 with or without 600 ng/ml doxycycline (Millipore) from day 4 EBs, as indicated. On day 7, all cultured cells were collected, and CD11b^−^CD45^+^ cells were sorted on FACS Melody (BD Biosciences) and plated in the methylcellulose for B-progenitor colony-forming assays (STEMCELL Technologies Methocult M3630) with 1×10^5^ OP9 cells, and with mIL7 10 ng/ml and mFlt3L 10 ng/ml. Seven to 10 days after plating, each colony was picked and stained using anti-AA4.1, -CD19 and -CD20 antibodies (all 1:200 dilution) for flow cytometric analysis. B1 progenitors were defined as AA4.1^+^CD19^+^B220^−^ cells; B2 progenitors were defined as AA4.1^+^CD19^−^B220^+^ cells, as previously described. For T cell analysis: DN, CD4-CD8^−^; DN1, CD4^−^CD8^−^CD44^+^CD25^−^; DN2, CD4^−^CD8^−^CD44^+^CD25^+^; DN3, CD4^−^CD8^−^CD44^−^CD25^+^; DP, CD4^+^CD8.

## Supplementary Material

Supplementary information

Reviewer comments
