# Supplementary material for: B1 lymphocytes develop independently of Notch signaling during mouse embryonic development
Source: Development. 2021 Aug 9;148(15):dev199373. doi: 10.1242/dev.199373 (PMC8380455; doi:10.1242/dev.199373)
Supplement: Supplementary information [file develop-148-199373-s1.pdf]

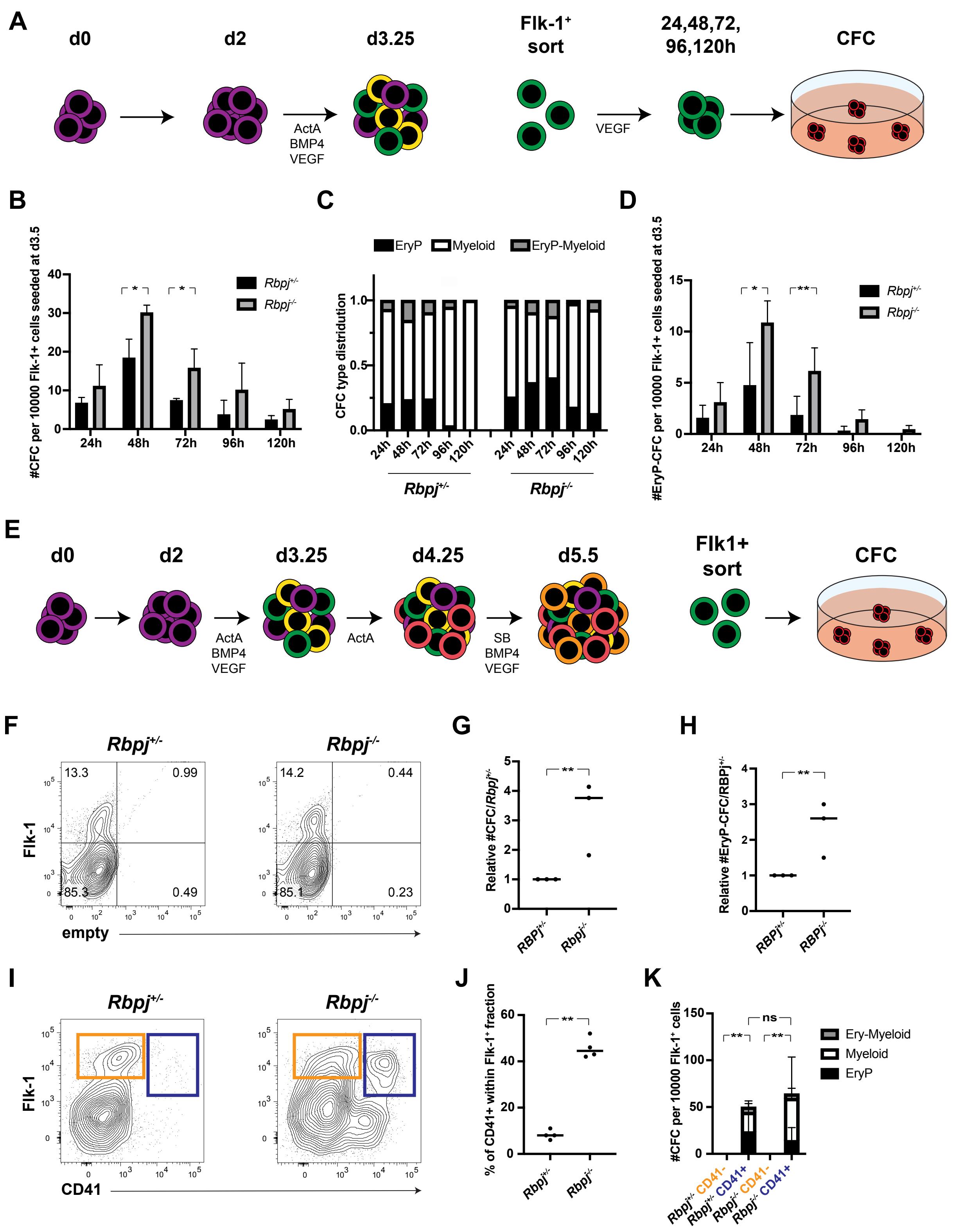

Development • Supplementary information

Fig. S1. Primitive erythropoiesis termination is impaired in the absence of Notch signaling. A, Experimental design for experiments with day 3.25 Flk-1+ cells. B, Kinetics of CFC development from Flk-1+-derived aggregates and their hematopoietic lineage distribution (C). D, Kinetics of EryP-CFC (EryP). E, Experimental design for experiments with day 5.5 Flk-1+ cells. F, Representative flow cytometric analysis of the Flk-1 expression in d5.5. EBs, of 3 independent experiments. G, Relative number of CFCs and (H) EryP-CFC generated from d5.5 Fk-1+ cells. I, Representative flow cytometric analysis of the Flk-1 and CD41 expression in d5.5. EBs. J, Quantification of CD41+ cells present within the Flk1+ population. K, Frequency of CFC within the CD41 fractions of Flk-1+ cells. n=3, independent experiments. Student's unpaired t-test *$p < 0.05$, **$p < 0.01$.

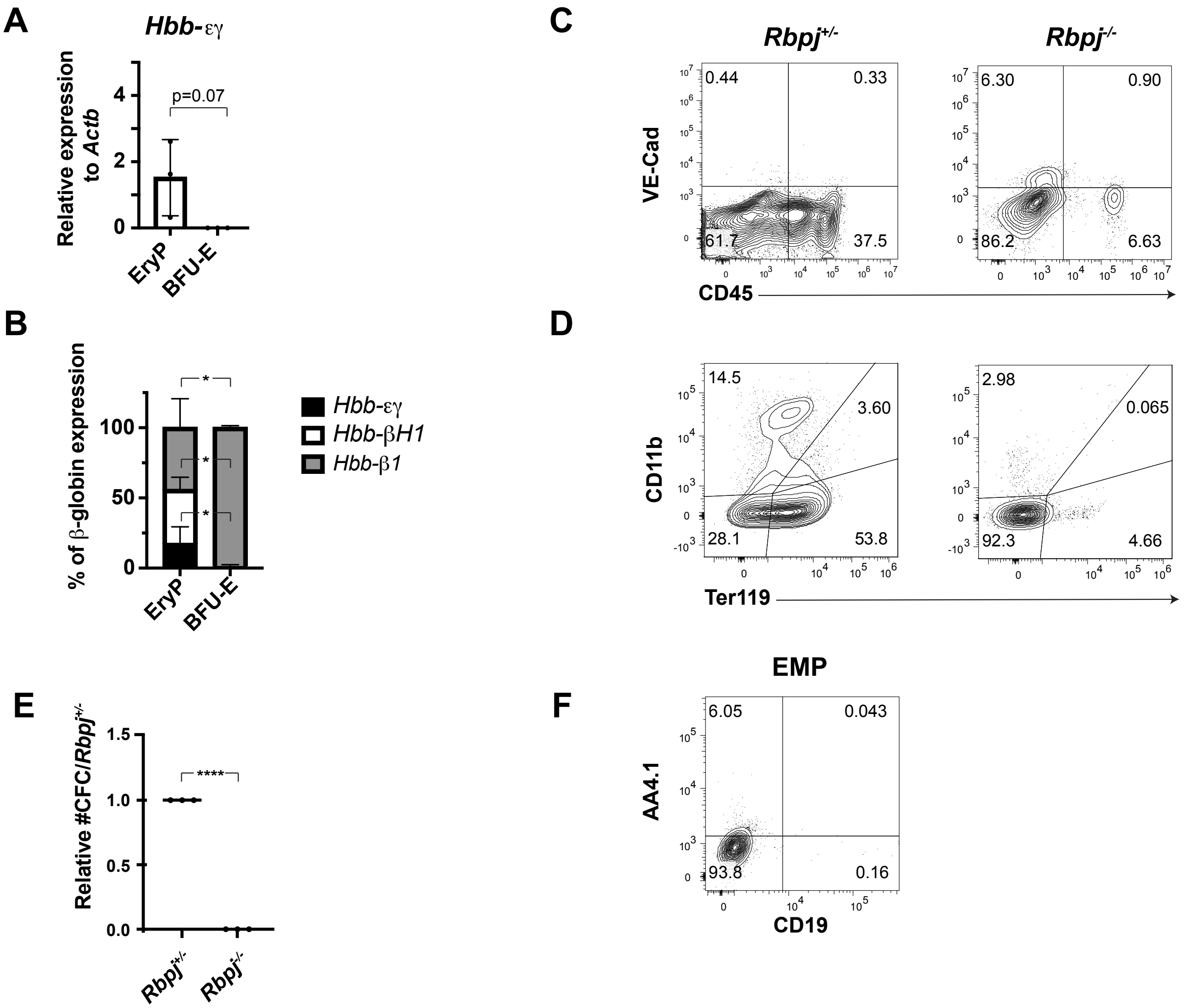

Fig. S2. Characterization of mESC- and YS-derived EMP hematopoietic output.

A, Real time qPCR analysis of the *Hbb-εγ* expression and (B) of the levels of εγ-, βH1-, and β1-globin transcripts graphed as a percentage of total β-globin transcripts in EryP-CFC (EryP) derived from day 3.25 Flk-1+ cells and BFU-E derived from day 5.5 Flk-1+ cells. C, Representative flow cytometric analysis of the VE-Cad and CD45 expression and (D) CD11b and Ter119 after 144 hours of EHT culture. E, Relative number of CFCs obtained after 144 hours of EHT culture. F, Representative flow cytometric analysis of the B-lymphoid lineage markers CD19 and AA4.1 after culture of cells isolated from E9.0-E9.5 YS of wild-type CD1 embryos as immunophenotypic EMPs for 10 days on OP9 stroma. n=3, independent experiments. Student's unpaired t-test $*p < 0.05$, $****p < 0.001$.

Development • Supplementary information
