# Supplementary material for: B1 lymphocytes develop independently of Notch signaling during mouse embryonic development
Source: Development. 2021 Aug 9;148(15):dev199373. doi: 10.1242/dev.199373 (PMC8380455; doi:10.1242/dev.199373)
Supplement: Reviewer comments [file develop-148-199373_review_history.pdf]

# B1 lymphocytes develop independently of Notch signaling during mouse embryonic development

Nathalia Azevedo Portilho, Rebecca Scarfò, Elisa Bertesago, Ismail Ismailoglu, Michael Kyba, Michihiro Kobayashi, Andrea Ditadi and Momoko Yoshimoto
DOI: 10.1242/dev.199373

**Editor**: Liz Robertson

**Review timeline**
Original submission: 21 December 2020
Editorial decision: 15 February 2021
First revision received: 17 May 2021
Accepted: 8 July 2021

**Original submission**

First decision letter

MS ID#: DEVELOP/2020/199373

MS TITLE: B-1 lymphocytes develop independently of Notch signaling during mouse embryonic development

AUTHORS: Nathalia Azevedo Portilho, Elisa Bertesago, Ismail Ismailoglu, Michihiro Kobayashi, Michael Kyba, Andrea Ditadi, and Momoko Yoshimoto

I have now received all the referees reports on the above manuscript, and have reached a decision. The referees' comments are appended below, or you can access them online: please go to BenchPress and click on the 'Manuscripts with Decisions' queue in the Author Area.

The overall evaluation is positive and we would like to publish a revised manuscript in Development, provided that the referees' comments can be satisfactorily addressed. Please attend to all of the reviewers' comments in your revised manuscript and detail them in your point-by-point response. If you do not agree with any of their criticisms or suggestions explain clearly why this is so.

We are aware that you may currently be unable to access the lab to undertake experimental revisions. If it would be helpful, we encourage you to contact us to discuss your revision in greater detail. Please send us a point-by-point response indicating where you are able to address concerns raised (either experimentally or by changes to the text) and where you will not be able to do so within the normal timeframe of a revision. We will then provide further guidance. Please also note that we are happy to extend revision timeframes as necessary.

Reviewer 1

*Advance summary and potential significance to field*

Innate immune B-1 B cells play important roles in mucosal immunity but their developmental origin is controversial. This paper provides evidence that B-1 cells emerge in the absence of Notch signaling. Since HSC are dependent upon Notch signaling for endothelial to hematopoietic transition

© 2021. Published by The Company of Biologists under the terms of the Creative Commons Attribution License (https://creativecommons.org/licenses/by/4.0/).

and generation of hematopoietic reconstitution, this novel information clarifies the Notch and HSC independent origin of transplantable B-1 cells. This is an important advance for understanding B-1 developmental origins.

*Comments for the author*

I have only a few questions for the authors:
Page 7 paragraph 1: "This reduction in hematopoietic output...." Was this reduction merely a delay in HEC production or a true deficiency state (if the cultures were extended beyond 96h would the total number of CFC still significantly differ)?
Readers need more explanation for how the authors identified B-1 from B-2 progenitor potential; how does the modified B-progenitor CFC assay discriminate?
An overall weakness of the paper is the lack of comparisons between embryos later than E9.5 from Rbpj+/- and -/- transgenic mice. Are there embryo survival issues that preclude examination of various B-1 and B-2 cells within the fetal liver?

Reviewer 2

*Advance summary and potential significance to field*

This manuscript addresses the question whether Notch signaling is required for B-1 cell production during mouse embryonic development, with the goal to use Notch dependence/independence as a proxy for HSC dependent and independent hematopoiesis. The authors use Notch signaling deficient mouse embryonic stem cell line (Rbpj-/- mESC), Rbpj-deficient mouse embryos, Notch signaling inhibition with DAPT, and Dox inducible Notch stimulation with NICD, in combination with various hematopoietic progenitor differentiation assays, to evaluate this question. The authors show that the development of B-1 lymphoid cells from mouse ES cells and in mouse embryos and yolk sacs can occur in the absence of Notch signaling, implying HSC independent origin. Their work also suggests that fine tuning the dosage of Notch signaling during mESC differentiation using Dox inducible NICD model influences B-1 vs B-2 cell specification from HEC precursors.
Notably, both B-1 and B-2 B cells increased in the presence of moderate Notch signaling stimulation, whereas only B-1 cells could be produced in complete absence of Notch activation. Further dissection between B-1a or B-1b cells was not conducted.

The present study is potentially interesting as it addresses the long-standing question of B-1 cell origin, and for the implications it can have for in vitro production of therapeutically valuable B-1 cells from PSCs. Since B-1 cells cannot be generated from BM HSCs, the findings from this manuscript are highly relevant for patients suffering from B-1 cell deficiency after BM transplantation, although it should be noted that mouse PSC differentiation to B lymphoid cells appears to be more robust than with human PSCs. Before this manuscript can be fully considered for publication the authors need to provide some key data points to support their conclusions, and put greater emphasis on the novel aspects of their study.

*Comments for the author*

Major Concerns

- This study shows that B-lymphocytes can develop independent of Notch signaling in mouse embryos and during ES cell differentiation, however, these data alone do not exclude the possibility that fetal liver HSCs or other Notch dependent precursors could also be a source of B-1a cells during development. Thus the model in which multiple precursors including fetal liver HSCs give rise to waves of B-1 cells during development (Baumgarth, J Immunol. 2017 and Montecino-Rodriguez Immunity 2012) cannot be rejected by the present data. This should be made clearer in the results/discussion section.
- Fig 3C shows that donor Rbpj-/- ESC-derived B cells reconstitute the B-1a compartment in the neonate NSG mice but fail to reconstitute the B-2 compartment. Why is Rbpj+/- control data not shown? How many mice were transplanted from each line? It is essential to validate that the control cells are capable of reconstituting both B-1 and B-2 compartments to ensure the assay is reliable to answer the question in hand.

© 2021. Published by The Company of Biologists under the terms of the Creative Commons Attribution License (https://creativecommons.org/licenses/by/4.0/). 

- It is clear from the ex vivo culture experiments with E9.5 YS and E9.5 P-Sp cells cultured on OP9 with or without gamma secretase inhibitor and from Cre+RbpjKO embryos that B-lymphopoiesis can occur independent of Notch signaling. However, the markers shown do not discern conclusively what type of B cells are produced in these experiments. It would be helpful to add additional markers such as CD5 and CD23 to distinguish between B-1a, B-1b, and B-2 cells.

- Although the finding that B-1 cells can develop in the absence of Notch signaling is important, the manuscript remains somewhat narrow in scope and advance since HSC independent origin of B-1 cells has already been suggested in prior studies, and the effects or lack of effects of Notch signaling on various other progenitors has been also studied extensively. It would be helpful if the authors could provide some information regarding the immunophenotype of the embryonic precursor that gives rise to B-1 cells , and at minimum determine if these precursors express the same or different surface phenotype as determined for EMPs (e.g. CD41+, ckit+, CD16/32+, CD45+/- etc). They should also document and discuss clearly the difference between B-1a, B-1b and B2 cells in their study. Better definition of the specific precursors for distinct B cell types would help direct future studies how to optimize the development of such precursors in vitro.

- It is not clear in the text and figure legends what population is considered as HEC in this study. Are sorted populations used for the culture assays when referred to HEC? It is also not clear how the authors justify using Ve-cadherin and CD45 as sole markers for EMP hematopoiesis from HEC. These have been classically used as markers for emerging HSCs in the AGM, whereas other markers have been commonly used for the identification of EMPs (CD41, ckit, CD16/32). Notably, not all EMP are CD45, especially during in vitro differentiation. It is thus important to clarify if the suggested reduction of EMP hematopoiesis in the absence of Notch signaling is a reflection of decreased expression of CD45, especially as prior studies have not noted a robust defect in EMP hematopoiesis in the absence of Notch signaling.

- Figure 4 is interesting and perhaps most important for the message and novelty of the manuscript, but the data is quite superficially presented and described. The authors note that moderate Notch stimulation using Dox inducible NICD cell line increases both B-1 and B-2 cells, although this data is not clearly shown in the figure. Please show the quantification of the different B cell types. Also, in Fig 4B, representative examples of B-1 and B-2 cells that form a single colony are shown. First, additional markers (e.g. CD5) would be helpful to confirm the B cell types (B-1a, B-1b, B-2). Also, it is not clear if B-1 and B-2 cells always segregate to separate colonies, or if there are colonies with mixed B cell types? This would be important to show. If mixed colonies are present, this would lead to different interpretation of the possible origins of B-1 cell precursors, and at least suggest that they may originate from multiple precursor types.

Minor Concerns and Comments

- Fig.1 How are EryP colonies evaluated as EryP vs EryD? No evaluation of hemoglobins being produced or colony morphology and hemoglobinization, are shown. Also, Fig.1 is largely a confirmation of previous studies that already document expansion of primitive hematopoiesis in Notch signaling deficient mouse ES cells differentiated in vitro, Moreover, this has not been observed robustly in vivo. As such, perhaps this data does not earn a full main figure for itself.

- How do the authors exclude the possibility that the kinetics of the development of various progenitors does not reflect natural variation between the mESC lines, rather than truly depend on Notch signaling?

- A simple schematic for each figure that depicts what model is used, what progenitor is being assayed and how the different cultures/differentiations are conducted and evaluated, would be helpful.

- On page 7 the authors state: “Collectively, these data show that Rbpj-/- mESCs can be used to dissect the Notch signaling requirement of different hematopoietic embryonic progenitors.” Since the in vitro differentiation protocols do not always fully recapitulate in vivo embryonic hematopoiesis (e.g. generate functional HSCs, among others) this statement should include the caveat that there may be discrepancies between in vivo and in vitro hematopoiesis.
- Fig2: the order of panels A, B and C in the figure does not match the figure legend.
- Description for Fig3 panel D is missing in the figure legend.

© 2021. Published by The Company of Biologists under the terms of the Creative Commons Attribution License (https://creativecommons.org/licenses/by/4.0/). 

- Fig4A: add quantification for the quadrants in CD44/CD25 plot.
- On page 8, in paragraph “The YS and P-Sp cells from Cre+RbpjKO and control embryos…” Fig. 1l is cited but it should be Fig3l.
- Are the YS and P-Sp cells taken from Cre+RbpjKO embryos at E9.5? It is neither stated in the text nor in the figures.
- There are several typos and minor language issues in the manuscript. Please proofread carefully to correct these, as well as the mislabelings in figures and figure citations.

---

**First revision**

Author response to reviewers' comments

Dear Reviewers

We would like to take the opportunity to thank both reviewers for the thoughtful comments and critiques. These have been helpful in our preparation of this revised manuscript, which includes additional experiments. We have also added extensive clarifications to many experiments and methods. Following the reviewers' advice, we have restructured the manuscript moving Fig. 1 to Fig. S1 and changed the figure numbers accordingly. In the revised manuscript, we have highlighted only major changes in blue font.

**Reviewer 1 Comments for the Author**: I have only a few questions for the authors:
Page 7 paragraph 1: "This reduction in hematopoietic output...." Was this reduction merely a delay in HEC production or a true deficiency state (if the cultures were extended beyond 96h would the total number of CFC still significantly differ)?
We acknowledge that the kinetics of the emergence of hematopoietic progenitors can differ across mESC lines. To address whether a delayed transition to hematopoiesis of day 5.5 Flk-1$^{+}$ *Rbpj*$^{-/-}$ cells could explain the observed results, we extended the differentiation cultures of day 5.5 Flk-1$^{+}$ from *Rbpj*$^{+/-}$ and *Rbpj*$^{-/-}$ mESCs up to 144h. At this time point, we have analyzed the presence of CD45$^{+}$ total hematopoietic cells, CD11b$^{+}$ myeloid cells, and Ter119$^{+}$ erythroid cells. We also quantified the frequency of erythro-myeloid progenitors present in the cultures with the classical methylcellulose-based clonogenic assay. The extended culture of day 5.5 Flk-1+ cells under these conditions amplified the differences between *Rbpj*$^{+/-}$ and *Rbpj*$^{-/-}$ cells initially observed at 96hrs for all these parameters. As such, our new data confirmed that the hematopoietic output from *Rbpj*$^{-/-}$ cells was not simply delayed, but diminished due to Notch deficiency. These results have been included in the revised manuscript (page 9) and Fig. S2C-E.

Readers need more explanation for how the authors identified B-1 from B-2 progenitor potential; how does the modified B-progenitor CFC assay discriminate?
Since B-cell progenitors generated *in vitro* from mESCs or *ex vivo* from mouse embryos do not express mature B-1 or B-2 cell markers (such as CD5 and CD23), the modified B-progenitor CFC assay allows to distinguish B-1 and B-2 progenitors based on their AA4.1, CD19, and B220 expressions. B-1 progenitors are defined as AA4.1$^{+}$CD19$^{+}$B220$^{-}$, while B-2 progenitors display an AA4.1$^{+}$CD19$^{-}$B220$^{+}$ phenotype (Montecino-Rodriguez et al., Nature immunology 2006). This assay has been previously described by the Dorshkind group (Montecino-Rodriguez et al. Immunity 2016), and we successfully used it to dissect the B-cell lineage potential of HSC precursors from the mouse embryo (Kobayashi et al., Stem Cell Reports 2019). In this manuscript, Flk-1$^{+}$ cells derived from mESCs were first seeded on OP9 stromal cells (in this specific case with different dosages of Doxycycline), in addition to mSCF, mIL-7, and mFlt-3L. Seven days later, CD11b$^{-}$CD45$^{+}$ hematopoietic progenitors were sorted from the co-cultures and plated onto methylcellulose with OP9 cells, mSCF, mIL-7, and mFlt3L. Each colony was picked and analyzed by flow cytometry for the expression AA4.1, CD19, and B220.
We have now added more details about the modified B-progenitor CFC assays in both the Methods

© 2021. Published by The Company of Biologists under the terms of the Creative Commons Attribution License (https://creativecommons.org/licenses/by/4.0/).

and Results sections (page 8 and 12-13).

An overall weakness of the paper is the lack of comparisons between embryos later than E9.5 from Rbpj+/- and -/- transgenic mice. Are there embryo survival issues that preclude examination of various B-1 and B-2 cells within the fetal liver?
As rightly guessed by the reviewer, unfortunately, the deletion of *Rbpj* in endothelial cells in VCCre$^{+}$:Rbpj$^{f/f}$ embryos induced embryonic lethality around E10. Therefore, it is not possible to analyze mouse embryos at stages later than the one we used for our experiments to provide the required comparison. We have added a sentence to clarify this point (page 11).

**Reviewer 2 Comments for the Author:** Major Concerns-This study shows that B-lymphocytes can develop independent of Notch signaling in mouse embryos and during ES cell differentiation, however, these data alone do not exclude the possibility that fetal liver HSCs or other Notch dependent precursors could also be a source of B-1a cells during development. Thus, the model in which multiple precursors including fetal liver HSCs give rise to waves of B-1 cells during development (Baumgarth, J Immunol. 2017 and Montecino-Rodriguez Immunity 2012) cannot be rejected by the present data. This should be made clearer in the results/discussion section.

We totally agree with the reviewer in that the results from our study do not exclude the possibility that other Notch-dependent progenitors, including fetal liver HSCs, could be an additional source of B-1a cells. Our objective was to provide formal proof that B-1 progenitors during development can be generated independently from HSCs. We used Notch-independency, a strict criterion for HSC-independent hematopoiesis, to address and validate our hypothesis, which is not mutually exclusive with a parallel generation of B-1a cells from HSCs. We have clarified this important point by adding a comment in our discussion (page 13).

-Fig 3C shows that donor Rbpj-/- ESC-derived B cells reconstitute the B-1a compartment in the neonate NSG mice but fail to reconstitute the B-2 compartment. Why is Rbpj+/- control data not shown? How many mice were transplanted from each line? It is essential to validate that the control cells are capable of reconstituting both B-1 and B-2 compartments to ensure the assay is reliable to answer the question in hand.

We agree with the reviewer in that we focused only on the data from *Rbpj*$^{-/-}$ cells, taking for granted the validation of our assay. In fact, we previously reported that the adoptive transfer of B-1 or B-2 progenitors from the fetal liver (FL) or BM reconstituted either mature B-1 or B-2 cells, respectively, in the recipient in NSG mice (Yoshimoto et al., PNAS 2011). Since the CD19$^{+}$B220$^{+}$ B-progenitor population derived *in vitro* can contain both B-1 and B-2 lineage progenitors, this adoptive transfer assay is a very useful method to determine and discriminate B-1 and B-2 lineage potentials. In the revised manuscript, we have now added the results from the transplant of FL mononuclear cells (MNCs, n=4) and *Rbpj*$^{+/-}$-derived B-cells (n=4) as controls. This is now shown in Fig. 2E. Of note, the mESC-derived B-cell progenitors derived in our settings are all HSC-independent and, as such, are devoid of B-2 lineage potential and unable to reconstitute the B-2 compartment in vivo, in contrast to FL MNCs. Collectively, these data indicate that the adoptive transfer is a valid assay to distinguish the B-cell lineage potential and shows that B-1 potential can arise in a Notch-independent manner.

-It is clear from the ex vivo culture experiments with E9.5 YS and E9.5 P-Sp cells cultured on OP9 with or without gamma secretase inhibitor and from Cre+RbpjKO embryos that B-lymphopoiesis can occur independent of Notch signaling. However, the markers shown do not discern conclusively what type of B cells are produced in these experiments. It would be helpful to add additional markers such as CD5 and CD23 to distinguish between B-1a, B-1b, and B-2 cells.

To test the B-cell potential of our candidate cells (either *in vitro* or *ex vivo*), we have used a widely used *in vitro* assay that is based on the coculture with OP9 stromal cells. While these conditions efficiently promote B-lymphopoiesis, it takes more than 4 weeks for hematopoietic progenitors, including YS/P-Sp-derived cells, to differentiate into IgM$^{+}$ mature B-cells. However, the expression of CD11b, CD5, or CD23 on B-cells is not properly induced in these cultures. Thus, under these conditions, the expression of the classical markers used to discriminate B-cell subsets cannot be

© 2021. Published by The Company of Biologists under the terms of the Creative Commons Attribution License (https://creativecommons.org/licenses/by/4.0/).

used. Furthermore, at the progenitor level, once AA4.1$^+$CD19$^+$B220$^-$ (CD19 single-positive) B-1 progenitors or AA4.1$^+$CD19$^-$B220$^+$ (B220$^+$ single-positive) B-2 progenitors mature into CD19$^+$B220$^+$ (double-positive) pre-B cells, there are no specific markers to distinguish B-1 from B-2 cells. Therefore, the best assays to evaluate B-1 or B-2 potential are: 1) the *in vivo* adoptive transfer assay, in which CD19$^+$B220$^+$ B-progenitors can differentiate into mature B-cell subsets in vivo, and 2) the modified B-progenitor CFC assays, which discriminate B-1 and B-2 progenitors on the basis of CD19 and B220 expression levels. Both assays are exploited in our manuscript for B-cell lineage characterization and our data collectively show that B-1 but not B-2 lineage potential is present in Flk-1$^+$-derived progenitors in the absence of Notch signaling.

-Although the finding that B-1 cells can develop in the absence of Notch signaling is important, the manuscript remains somewhat narrow in scope and advance since HSC independent origin of B-1 cells has already been suggested in prior studies, and the effects or lack of effects of Notch signaling on various other progenitors has been also studied extensively. It would be helpful if the authors could provide some information regarding the immunophenotype of the embryonic precursor that gives rise to B-1 cells, and at minimum determine if these precursors express the same or different surface phenotype as determined for EMPs (e.g. CD41+, ckit+, CD16/32+, CD45+/- etc). They should also document and discuss clearly the difference between B-1a, B-1b and B2 cells in their study. Better definition of the specific precursors for distinct B cell types would help direct future studies how to optimize the development of such precursors in vitro.

Notch signaling has been indeed extensively characterized during hematopoietic development. However, most of the characterization of Notch-independent hematopoiesis has focused on the emergence of erythroid and myeloid cells and the impact of Notch signaling on the generation of specific subsets of hematopoietic cells remains poorly studied.

While it is widely accepted that the fine-tuning of Notch signaling in endothelial cells regulates the emergence of HSCs, whether specific lymphoid precursors emerge from endothelial cells in the absence of Notch-signaling remains unknown. While T- and B- lymphoid potential is detectable as early as E8.25, at a pre-circulation stage, using organ culture or co-culture system, whether this earliest lymphoid potential represents HSC-independent hematopoiesis or the precursor of HSCs that are not detectable by transplantation assays is still a matter of debate. Therefore, validating the presence of HSC-independent B-lymphopoiesis is critically important to understand the multiple waves of hematopoiesis that occur during the embryonic period

In addition, the lack of understanding of the development of specific blood cell types hampers efforts to generate them *in vitro* for therapeutic purposes. Given the role of B-1 cells in mediating protection against atherosclerosis and other inflammatory diseases and the inability of adult HSCs to reconstitute the B-1 cell compartment upon bone marrow transplant, a better understanding of the ontogeny of this important subset of immune cells is needed. Therefore, our studies proving that B-1 cells can be efficiently generated in the absence of Notch signaling are critical for designing strategies to derive B-1 cells *in vitro*, in particular from pluripotent stem cells. Therefore, we believe that, while focused on a very precise question, the importance of our results can expand beyond the field of hematopoietic development with potential implications for cell therapies and regenerative medicine.

B-1 specific precursors in the embryo have been the subject of intense investigation for decades, but they are yet to be precisely identified. Nonetheless, in previous studies, we and others have found that neither YS-derived EMPs, CD41$^+$ nor CD45$^+$ hematopoietic progenitors of E9.5 embryos can produce B-cells *ex vivo* (Nishikawa et al., Immunity 1998; Yoshimoto et al., PNAS 2011; McGrath et al., Cell Reports 2015). Instead, only endothelial cells have lymphoid producing capacity at this stage. In this study, we reconfirmed that the major HSC-independent progenitor cells, *i.e.*, EMPs, isolated from E9.5 wild-type YS cannot produce B-cells *in vitro* (data shown in Fig. S2F). Thus, it is highly unlikely that EMPs represent the progenitors of Notch-independent B-1 cells and B-1 cell lineage specification seems to occur specifically in the endothelial cells.

-It is not clear in the text and figure legends what population is considered as HEC in this study. Are sorted populations used for the culture assays when referred to HEC? It is also not clear how the authors justify using Ve-cadherin and CD45 as sole markers for EMP hematopoiesis from HEC. These have been classically used as markers for emerging HSCs in the AGM, whereas other markers

© 2021. Published by The Company of Biologists under the terms of the Creative Commons Attribution License (https://creativecommons.org/licenses/by/4.0/).

have been commonly used for the identification of EMPs (CD41, ckit, CD16/32). Notably, not all EMP are CD45, especially during in vitro differentiation. It is thus important to clarify if the suggested reduction of EMP hematopoiesis in the absence of Notch signaling is a reflection of decreased expression of CD45, especially as prior studies have not noted a robust defect in EMP hematopoiesis in the absence of Notch signaling.

We agree with the reviewer that we have not properly characterized hemogenic endothelial cells (HECs). In this current version of the manuscript, we have carefully revised the language used to describe our candidate populations. Throughout our mESC differentiation studies, we have evaluated the B-lymphoid potential of Flk-1$^{+}$ cells. While this population clearly contains HECs and/or HEC precursors, not all the Flk-1$^{+}$ cells isolated possess hematopoietic potential.

In particular, day 5.5 Flk-1$^{+}$ cells generate hematopoietic progenitors whose potential resemble that of YS-derived EMP progenitors, as shown by Clarke and colleagues (Stem Cell Reports 2015). Here, we are confirming that upon an endothelial-to-hematopoietic transition (EHT), day 5.5 Flk-1$^{+}$ cells generate BFU-E whose progeny express much lower levels of *Hbb-εy* compared to EryP-CFC as they express mostly *Hbb-β1*, similarly to EMP in culture. We, therefore, used a functional definition for EMPs in our studies.

We also agree with the reviewer that not all the EMP emerging in the YS are CD45$^{+}$. In this study, we evaluated functional EMP production as erythroid and myeloid colony forming cells, thus, the total cultured cells were seeded in the clonogenic assay regardless of CD45 expression to monitor the impact of the absence of Notch signaling in the hematopoietic output from day 5.5 Flk-1+ cell. Of note, we monitored VE-Cadherin and CD45 expression as a functional readout of the capacity of day 5.5 Flk-1$^{+}$ cells to undergo EHT. It has been reported that in the absence of Notch signaling, the emergence of blood cells from ECs in the AGM is severely impaired, while EMPs are produced from YS ECs (Kumano et al, Immunity 2003, Robert-Moreno et al., Development 2005). Similarly, day 5.5 *Rbpj*$^{-/-}$ Flk-1$^{+}$ cells were able to complete the EHT and generate CD45$^{+}$ hematopoietic cells, although the proportion of CD45$^{+}$ cells observed was always lower than in *Rbpj*$^{+/-}$ cultures. We have now added new data in Figure 2C-E, showing that upon prolonged culture (up to 144h), these differences in hematopoietic output (measured as a proportion of CD45$^{+}$, CD11b$^{+}$, and Ter119$^{+}$ as well as clonogenic progenitors) further increased. Collectively, our results suggest that while not required for the emergence of EMPs during embryonic development, Notch signaling might play an unexpected role during the proliferation and/or survival of these hematopoietic progenitors or their downstream progeny. EMP-specific deletion of Notch signaling has not been reported yet, limiting our ability to understand the exact role of Notch during different phases of hematopoietic differentiation for this specific developmental program. We believe that our results are in line with what Hadland and colleagues (Blood 2004) have shown *in vivo* by using Notch1$^{-/-}$ chimeric mice. In fact, they reported a 10-fold reduction of clonogenic progenitors in Notch1$^{-/-}$ E13.5 FLs, a stage where both HSC-dependent and HSC-independent progenitors are present. In their settings, it is difficult to dissect whether the observed reduction is due to the absence of HSCs, the impaired proliferation of EMPs and other Notch-independent progenitors, or both. Our mESC-based approach using specific protocols to independently study primitive- or EMP-like hematopoiesis provides an additional and useful tool to dissect the regulation of specific embryonic hematopoietic programs. We have discussed this point on page 9.

-Figure 4 is interesting and perhaps most important for the message and novelty of the manuscript, but the data is quite superficially presented and described. The authors note that moderate Notch stimulation using Dox inducible NICD cell line increases both B-1 and B-2 cells, although this data is not clearly shown in the figure. Please show the quantification of the different B cell types. Also, in Fig 4B, representative examples of B-1 and B-2 cells that form a single colony are shown. First, additional markers (e.g. CD5) would be helpful to confirm the B cell types (B-1a, B-1b, B-2). Also, it is not clear if B-1 and B-2 cells always segregate to separate colonies, or if there are colonies with mixed B cell types? This would be important to show. If mixed colonies are present, this would lead to different interpretation of the possible origins of B-1 cell precursors, and at least suggest that they may originate from multiple precursor types.

© 2021. Published by The Company of Biologists under the terms of the Creative Commons Attribution License (https://creativecommons.org/licenses/by/4.0/).

While the quantification of the different B-cell types is shown in current Fig. 4D (Fig. 4C in the previous version), we agree that its interpretation is difficult. The modified B-cell colony assay was described in our previous report (Kobayashi et al., Stem Cell Reports 2019), where we successfully used it to dissect the B-cell lineage potential of HECs isolated from mouse embryos. To clarify the specific point raised by the reviewer, the presence of B-2 progenitors was always observed in colonies containing also B-1 progenitors. In the absence of Notch signaling activation via Dox administration, colonies generated by mESC-derived CD11b$^-$CD45$^+$ cells are composed uniquely by B-1 progenitors. On the other hand, the activation of Notch signaling increased the number of colonies that contain both B-1 and B-2 progenitors. We have clarified this in the revised manuscript.

Furthermore, the fact that Notch activation in Flk-1$^+$-derived precursors expands their B-cell potential to include the B-2 cell lineage, can be interpreted in different ways:

1) Notch activation elicits an HSC program in Flk-1$^+$ cells;
2) B-1 progenitors may be generated in an HSC-independent manner through a B-1/B-2 bipotent progenitor, a common lymphoid progenitor or a multipotent progenitor intermediate, whose B-cell fate choice is regulated by Notch signaling.

Both these scenarios are supported by our previous reports in which:
1) single immunophenotypic pre-HSCs, which display a B-1 biased potential, can be coaxed *in vitro* to develop into multilineage repopulating cells (including B-1a, B-1b, B-2, and T-lymphoid potential) upon co-culture with Notch expressing AGM-derived endothelial cells (Hadland et al., Stem Cell Reports 2017). This suggests that Notch may expand the lineage potential in hematopoietic stem/progenitor cells.
2) a subset of immunophenotypic pre-HSC can reconstitute only the B-1 and T-cell compartments upon direct transplant (Kobayashi et al., Stem Cell Reports 2019). This suggests the existence of HSC-independent B-1 and T-lymphoid progenitors.
We have discussed these possibilities on page 13 in the revised manuscript.

Importantly, this assay can only detect progenitors endowed with B-1 or B-2 lineage potential and is not permissive for the growth of mature B-1a/B-1b/B-2 cells. As such, these B-1/B-2 progenitors in the colonies do not express CD5 or CD23, which are not reliable and useful markers for *in vitro* or *ex vivo*-derived B-cells, as discussed previously. Therefore, it is not possible to further discriminate between different B-cell lineages. However, combined with our adoptive transfer assay, these results clearly indicate that B-cells of the B-1 lineage can be derived in the absence of Notch signaling.

Minor Concerns and Comments-Fig.1 How are EryP colonies evaluated as EryP vs EryD? No evaluation of hemoglobins being produced, or colony morphology and hemoglobinization, are shown. Also, Fig.1 is largely a confirmation of previous studies that already document expansion of primitive hematopoiesis in Notch signaling deficient mouse ES cells differentiated in vitro, Moreover, this has not been observed robustly in vivo. As such, perhaps this data does not earn a full main figure for itself.

A thorough characterization of the erythroid progenitors generated using the protocols for mESC hematopoietic differentiation, which we have adopted in our studies, was already provided by Clarke and colleagues (Stem Cell Reports 2015). There, the authors showed clearly that day 3.25 Flk-1$^+$ progenitors give rise to almost exclusively EryP-CFC expressing high levels of *Hbb-εy*, while upon EHT culture day 5.5 Flk1$^+$ progenitors generate BFU-E that are reminiscent of those derived from YS-derived EMPs. As mentioned above, we are now providing data that confirm these protocols yield Flk-1$^+$ at day 3.25 that are enriched for primitive progenitors, while day 5.5 Flk-1$^+$ cells are correlatives of YS EMPs. We have in fact analyzed via quantitative PCR the *β*- globin content of EryP-CFC derived from day 3.25 Flk-1$^+$ cells and of BFU-E generated from day 5.5 Flk-1$^+$ cells that underwent EHT. We find that EryP-CFC expresses higher levels of *Hbb-εy* compared to BFU-E, where transcripts for this isoform are barely detected as they express mostly *Hbb-β1*. These data are now shown in Fig. S2A, B.
In addition, we agree with the reviewer that our data on primitive hematopoiesis validate the model but does not provide additional knowledge. Therefore, as suggested, in this revised version, we have moved Fig.1 to Fig. S1.

© 2021. Published by The Company of Biologists under the terms of the Creative Commons Attribution License (https://creativecommons.org/licenses/by/4.0/).

-How do the authors exclude the possibility that the kinetics of the development of various progenitors does not reflect natural variation between the mESC lines, rather than truly depend on Notch signaling?

The reviewer raises an important point regarding mESC line variability, which we also discuss in our comment#1 to reviewer 1.
We have addressed this point in two ways:
1) by carefully analyzing a broad window of time points when we have observed major differences in assays performed with mESC-derived progenitors. This was the case already for the characterization of the primitive wave, using day 3.25 Flk-1$^+$ cells (shown in old Fig. 1A-C, now Fig S1A-C). We are now providing new data from experiments performed with day 5.5 Flk-1$^+$ progenitors (now present in Fig. S2C-E), where we extended the EHT cultures up to 144h. At this time point, *Rbpj*$^{-/-}$ cells display a much greater impairment in their hematopoietic output, measured as a proportion of CD45$^+$ hematopoietic cells, CD11b$^+$ myeloid cells, and Ter119$^+$ erythroid cells as well as clonogenic progenitors, indicating that these differences are not due to delayed kinetics but are rather an outcome of Notch deficiency.
2) performing mouse embryo experiments in parallel to confirm the hematopoietic output of various progenitors is similar between the mouse embryo and mESCs. Our data indicate that what we observed in the Notch-independent mESC cultures (presence of B-1 potential) is similar to Notch-independent YS/P-SP-derived hematopoietic development.

-A simple schematic for each figure that depicts what model is used, what progenitor is being assayed and how the different cultures/differentiations are conducted and evaluated, would be helpful.

We have now added a descriptive schematic for all our experimental designs.

-On page 7 the authors state: "Collectively, these data show that Rbpj-/- mESCs can be used to dissect the Notch signaling requirement of different hematopoietic embryonic progenitors." Since the in vitro differentiation protocols do not always fully recapitulate in vivo embryonic hematopoiesis (e.g. generate functional HSCs, among others) this statement should include the caveat that there may be discrepancies between in vivo and in vitro hematopoiesis.

We agree with the reviewer in that, in the absence of robust *in vitro* generation of HSCs, the mESC differentiation system does not faithfully recapitulate the entire murine embryonic hematopoietic development, but only the HSC-independent hematopoietic developmental waves. We have commented on this important point in our discussion (page10).

-Fig2: the order of panels A, B and C in the figure does not match the figure legend.

We have corrected the figure legend in our revised manuscript.

-Description for Fig3 panel D is missing in the figure legend.

We have now added the legend for panel D.

-Fig4A: add quantification for the quadrants in CD44/CD25 plot.

We have now added the quantification for CD44/CD25 flow cytometry analysis in Fig. 4B

-On page 8, in paragraph "The YS and P-Sp cells from Cre+RbpjKO and control embryos…" Fig. 1l is cited, but it should be Fig3l.

We have now corrected the text.

-Are the YS and P-Sp cells taken from Cre+RbpjKO embryos at E9.5? It is neither stated in the text nor in the figures.

© 2021. Published by The Company of Biologists under the terms of the Creative Commons Attribution License (https://creativecommons.org/licenses/by/4.0/).

As the reviewer rightly guessed, YS and P-Sp were harvested from E9.5 *Cre*$^{+}$*RbpjKO* embryos. We have now added this information in the text and figure legends.

-There are several typos and minor language issues in the manuscript. Please proofread carefully to correct these, as well as the mislabelings in figures and figure citations.
We apologize. We have corrected typos and mislabeling and sent the manuscript for proofreading.

---

Second decision letter

MS ID#: DEVELOP/2020/199373

MS TITLE: B-1 lymphocytes develop independently of Notch signaling during mouse embryonic development

AUTHORS: Nathalia Azevedo Portilho, Rebecca Scarfo, Elisa Bertesago, Ismail Ismailoglu, Michael Kyba, Michihiro Kobayashi, Andrea Ditadi, and Momoko Yoshimoto

ARTICLE TYPE: Research Report

I am happy to tell you that your manuscript has been accepted for publication in Development, pending our standard ethics checks.

Reviewer 1

*Advance summary and potential significance to field*

The authors have clearly demonstrated that B-1 B cell progenitors are not derived via precursors (HSC) dependent upon Notch signaling. Yet, the B-1 cells engraft in murine hosts indicative of display of the properties of engraftment, migration, and expansion. Thus, certain B cell precursors are derived prior to fetal liver HSC expansion (Notch dependent) and populate the host long-term.

*Comments for the author*

The authors have addressed the primary concerns of this reviewer. The revised paper is clearly improved.

Reviewer 2

*Advance summary and potential significance to field*

The authors have done extensive revisions, including new experiments and clarification of the data presentation and message. This work now shows conclusively the Notch1 independent origin of B-1 B cells whereas B-2 cell development is Notch dependent.

*Comments for the author*

I am satisfied with the responses to my concerns.

© 2021. Published by The Company of Biologists under the terms of the Creative Commons Attribution License (https://creativecommons.org/licenses/by/4.0/).
